# *In Vivo* Anti-*Trypanosoma cruzi* Activity of Hydro-Ethanolic Extract and Isolated Active Principles from *Aristeguietia glutinosa* and Mechanism of Action Studies

**DOI:** 10.3390/molecules19068488

**Published:** 2014-06-23

**Authors:** Javier Varela, Elva Serna, Susana Torres, Gloria Yaluff, Ninfa I. Vera de Bilbao, Patricio Miño, Ximena Chiriboga, Hugo Cerecetto, Mercedes González

**Affiliations:** 1 Grupo de Química Medicinal-Laboratorio de Química Orgánica-Facultad de Ciencias-Facultad de Química—UdelaR, Iguá 4225, Montevideo, C.P. 11400, Uruguay; E-Mail: jvarelaubillos@gmail.com; 2 Departamento de Medicina Tropical, Instituto de Investigaciones en Ciencias de la Salud, Universidad Nacional de Asunción, Asunción, 1120, Paraguay; E-Mails: elvsern@hotmail.com (E.S.); susitorres1@hotmail.com (S.T.); gloriayaluff@yahoo.com (G.Y.); nverabilbao@gmail.com (N.I.V.B.); 3 Carrera Química Farmacéutica, Facultad de Ciencias Químicas, Universidad Central de Ecuador, Quito, C.P. 170150, Ecuador; E-Mails: patriciomino@yahoo.com (P.M.); xchiriboga@yahoo.com (X.C.)

**Keywords:** anti-*T. cruzi* activity, *Aristeguietia glutinosa* Lam., (+)-15-hydroxy-7-labden-17-al, labdane diterpenoid, ^1^H-NMR metabolomics

## Abstract

The currently available treatments for Chagas disease show limited therapeutic potential and are associated with serious side effects. Attempting to find alternative drugs isolated from Nature as agents against *Trypanosoma cruzi* has been our goal. Recently, we have demonstrated the *in vitro* anti-*T. cruzi* activities of two secondary metabolites isolated from the hydro-ethanolic extract of the aerial parts of *Aristeguietia glutinosa* (Lam.), (family Asteraceae). These active principles displayed poor hemolytic activity, low toxicity against murine macrophages, and absence of mutagenicity. Herein, proof of concept *in vivo* studies of the whole hydro-ethanolic extract of the aerial parts of *Aristeguietia glutinosa* and of the most active component isolated from the hydro-ethanolic extract, *i.e.*, (+)-15-hydroxy-7-labden-17-al, was done in a murine acute model of Chagas disease. Both treatments caused a decrease in the animals’ parasitemia. Metabolomic mechanism of action studies were done by ^1^H-NMR, both on the extract and on the active compounds, examining the effects of the metabolites both on membrane sterol biosynthesis and mitochondrial dehydrogenases, whereby we found that one of the metabolites inhibited the activity of the parasite mitochondrial dehydrogenases and the other inhibited the biosynthesis of parasite membrane sterols. The results are interesting in the context of popular use of plants for the treatment of Chagas disease.

## 1. Introduction

Chagas disease is caused by the flagellate protozoan *Trypanosoma cruzi* (*T. cruzi*). It is transmitted to humans by bites and concomitant defecation of different triatomine species, which carry the parasite in their contaminated feces. Other modes of transmission include blood transfusion or blood infection from infected mother to her child, or by oral ingestion of food contaminated with parasites [1]. It is an endemic disease that affects millions of people generating health, economic and social problems in the countries affected [2]. It is widespread in Central and South America, affecting 21 countries in these regions. It has been estimated that the disease affects between 9.8 and 11.0 million people, while 60.0 millions are at risk. This is due to both population mobility between Latin America and the rest of the world or residence in endemic areas.

Despite the time elapsed since the Brazilian doctor Carlos Ribeiro Justiniano Chagas discovered the disease in 1909, still there are no effective chemotherapies for all its clinical forms [3]. Like other neglected diseases, is a major health problem as a result of inadequate therapy and the lack of an effective vaccine [4]. Research work in this medical area in order to find new solutions to a problem that seems to have no end is therefore of utmost importance [5].

**Figure 1:**
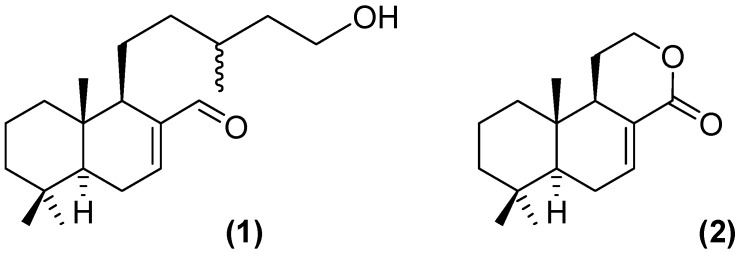
Structures of the most active anti-*T. cruzi* compounds isolated from whole hydro-ethanolic extract of *A. glutinosa* Lam. [16]: (+)-15-hydroxy-7-labden-17-al (**1**) and (+)-13,14,15,16-tetranorlabd-7-en-l7,12-olide (**2**).

The genus *Aristeguietia* (family Asteraceae) consists of twenty one species distributed on the Andes from Colombia to south Peru [6,7]. Plants of this genus have been used in traditional medicine for many decades and bioactive natural products have been isolated that could be promising bioresources for the preparation of drugs [8,9]. In Ecuador nine Andean species have been described; among them *Aristeguietia* *glutinosa* (Lam.) [6], traditionally known by the popular names matico, yerba del soldado, chuzalongo, matigo, or migla, according to the region, grows at an altitude of about 3,000 m and its leaves and twigs decoction has been used as an astringent, antirheumatic, antimicrobial, anti-stomach ulcers, and to treat diarrhea, and headaches [10,11]. Some phytochemical and pharmacological studies on *A. glutinosa* Lam. have been reported, identifying compounds with antimicrobial [12,13], and antiviral activities [14]. As part of our ongoing pharmacological studies of Andean-Ecuadorian plants we have analyzed, among others, the whole hydro-ethanolic extract from aerial parts (WHEAP) of *A. glutinosa* against bacteria (*B. subtilis*, *S. epidermidis*, *S. aureus*, *E. coli*, *P. aeruginosa*, *K. pneumoniae*, and *S. typhi*) and fungi (*T. rubrum*, and *M. canis*) finding excellent antifungal activity on the same order of the control (griseofulvin) [15]. Additionally, we have recently reported the bioactive-guided identification of two labdane diterpenoids from aerial parts of *Aristeguietia glutinosa* Lam. as *in vitro* anti-*T. cruzi* agents [16]. The WHEAP showed anti-*T. cruzi* activity against epimastigotes (IC_50_ = 19.6 µg/mL), whereas the isolated compounds (+)-15-hydroxy-7-labden-17-al (**1**, Figure 1) and (+)-13,14,15,16-tetranorlabd-7-en-l7,12-olide (**2**, Figure 1) were nearly seven- and one a half-fold, respectively (IC_50_ = 3.0 and 15.6 µg/mL or 9.8 and 62.9 µM), more active than the original extract while showing also both a lack of mammalian cytotoxic and mutagenic effects, whereas the clinically used nifurtimox (Nfx, IC_50_ = 7.7 µM [16]), equipotent to compound **1**, is mutagenic [16]. This could at least support the vernacular medicinal use of *A. glutinosa* Lam. as an anti-Chagas agent.

The present study was conducted in order to prove the concept, for *in vivo* anti-*T. cruzi* evaluation of active principles and the whole plant extract from *Aristeguietia glutinosa* and to attempt to elucidate the possible mechanism(s) of action.

## 2. Results and Discussion

### 2.1. Proof of Concept

Firstly, we studied the effects of the WHEAP and isolated compounds from the different fractions against the bloodstream trypomastigote form of *T. cruzi* at 250 µg/mL and at 4 °C (Table 1) [17]. The results allowed us to propose the WHEAP and compound **1** as potential agents to eradicate trypomastigotes in contaminated blood from transfusion-blood-banks; we discarded compound **2** from further *in vivo* studies, due its lower *in vitro* effects against this form, and selected the WHEAP and compound **1** for further *in vivo* studies.

**Table 1:** Results of the studies against bloodstream, trypomastigote, form of *T. cruzi* (CL Brener clone). The extract, pure compounds and GV were studied at 250 µg/mL. The experiments were done in duplicate.

Extract or Compound	Percentage of Lyses (%) ^a^
WHEAP	82 ± 5
(**1**)	91 ± 4
(**2**)	54 ± 7
GV ^b^	90 ± 6

^a^ Respect to untreated parasites; ^b^ GV: gentian violet (reference trypanosomicidal agent).

**Figure 2:**
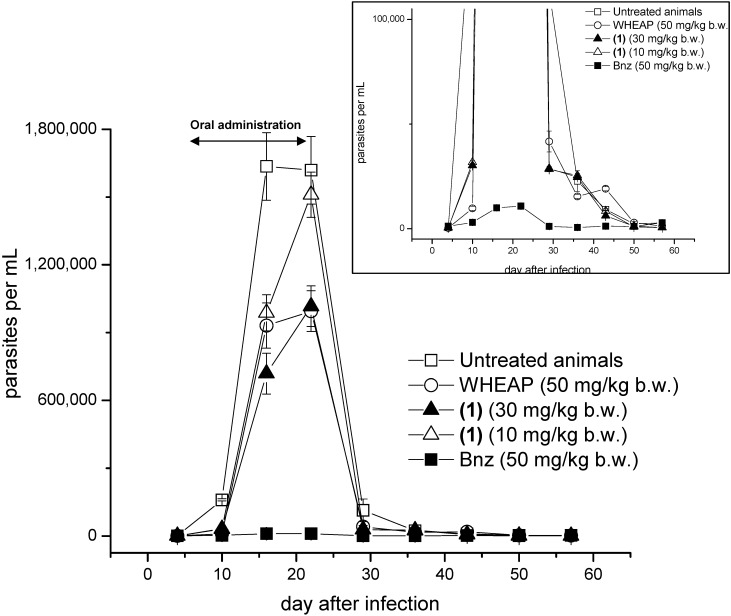
Parasitemia *versus* days post-infection in the *in vivo* activity assays (the oral administration was done between days 7 and 21). Eight animals were used in each experimental group. Inset: enlargedregion showing the behavior of Bnz.

Secondly, the *in vivo* efficacy was evaluated in a murine acute model of Chagas disease. BALB/c mice were infected intraperitoneally with 5,000 trypomastigotes of CL Brener clone and when the parasitemia was established (7th day) the oral treatments began for the consecutive 14 days [17]. For the wheap a dose of 50 mg/kg b.w. was used, while for the active compound **1** two different doses were employed, 10 and 30 mg/kg b.w. During the infection with CL Brener clone, the maximum peak of parasitemia was at 16th day postinfection with 16.4 × 10^5^ parasites per mL of blood, while for the studied compounds and the reference drug, benznidazole (Bnz), the maximum peaks of parasitemia were at 22nd day postinfection with 9.9 × 10^5^ for WHEAP, 10.2 × 10^5^ for **1** at 30 mg/kg b.w., 15.1 × 10^5^ for **1** at 10 mg/kg b.w., and 0.1 × 10^5^ for Bnz (Figure 2). 

**Figure 3:**
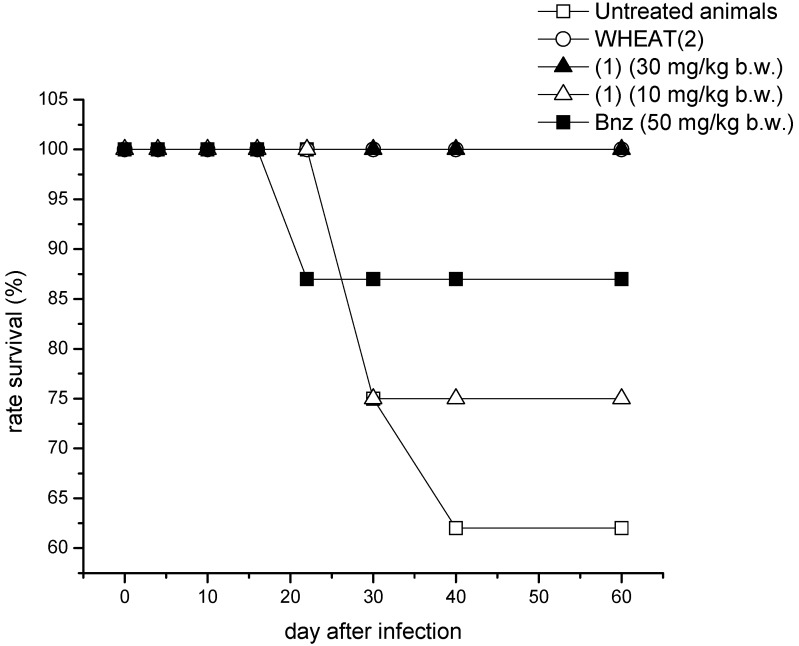
Survival rates in the *in vivo* activity assays. Eight animals were used in each experimental group.

These shifts in the day of the maximum peaks of parasitemia showed the effect of the studied compounds on the parasitemia load falling during the days of the treatment. At the same time a clear dose response was observed for (+)-15-hydroxy-7-labden-17-al (**1**), being the amount of parasites in blood significantly lower when the dose was 30 mg/kg b.w. According to the animal behavior, *i.e.*, changes in the plasticity, mobility, or food uptake, the ethanolic extract and the most active principle isolated from *Aristeguietia glutinosa*, *i.e.*, compound **1**, were well tolerated by the mice and no secondary side effects were observed in any of the experiments. Additionally, the studied extract and fraction allowed major animal survival rates than the untreated animals (Figure 3).

### 2.2. Accessing the Mechanism of Action

#### 2.2.1. ^1^H-NMR Metabolomic Studies

**Figure 4:**
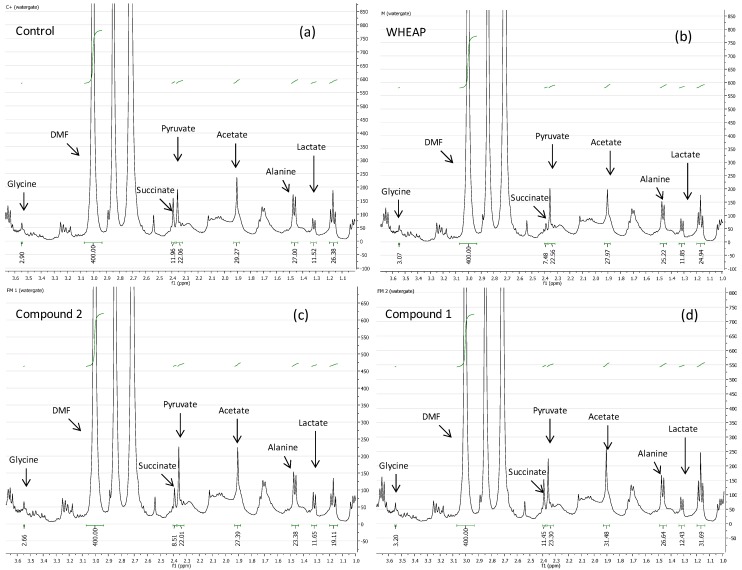
Examples of ^1^H-NMR spectra of the cell-free milieu of: (**a**) untreated parasite “control”; (**b**) wheap-treated parasites; (**c**) compound 2-treated parasites; (**d**) compound **1**-treated parasites.

**Figure 5:**
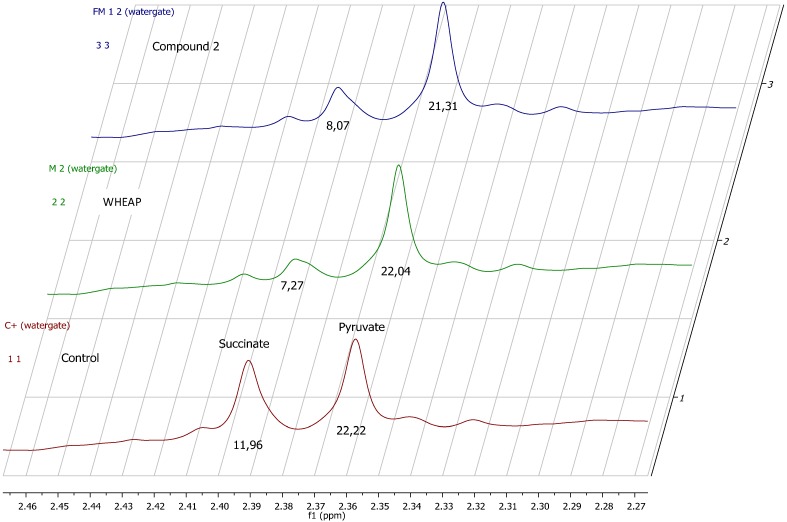
Stacked ^1^H-NMR spectra in the Succ and Pyr zone. A decrease in the Succ signal was clearly observed when the parasites were incubated with WHEAP or with compound **2**.

To gain insight into the mechanism of action the changes in biochemical pathways promoted by the active principles isolated from *Aristeguietia glutinosa* were studied by analyzing the metabolites excreted by the parasite by ^1^H-NMR spectroscopy. Changes in *T. cruzi* Y strains’ excreted metabolites when the parasite cells were exposed to a bioactive compound could be indicative of the biochemical pathway(s) modified by the agents [17,18,19,20]. The spectra of the cell-free milieu of treated parasites were compared with those of the untreated *T. cruzi* free milieu as the control. We focused mainly on the changes of the excreted salts of the carboxylic acids, lactate (Lac), acetate (Ace), pyruvate (Pyr), and succinate (Succ) and the amino acids, alanine (Ala) and glycine (Gly), among the most relevant modified metabolites. The studies revealed interesting results, as shown in Table 2 and Figure 4 and Figure 5. Clearly, wheap and compound **2** decreased the levels of excreted Succ, Ace, and Ala. This showed that wheap shared the same metabolic profile as compound **2**. However, compound **1** increased the level of excreted Ace. According to the *T. cruzi* metabolic pathways the changes in Succ and Ace, in the case of wheap and labdane **2**, could indicate that some enzymes in the mitochondria, like fumarate reductase, could be potential targets of these compounds [21]. The Ala-decreasing effect observed with wheap and labdane **2** could be the result of an inhibition of the cytosolic alanine aminotransferase that converts Pyr in Ala [22], so probably, compound **1** had a different mechanism of action or additional targets.

**Table 2:** Results in metabolomic studies using ^1^H-NMR (for details see Experimental Section). Each run was done in triplicate.

Compound ^a^/Metabolite ^b^	Gly	Succ	Pyr	Ace	Ala	Lac
wheap	2.96 ± 0.02	**7.27 ± 0.09** ^c^	22.04 ± 0.54	**27.39 ± 0.67**	**24.78 ± 0.38**	11.81 ± 0.01
( **1**)	3.07 ± 0.03	11.96 ± 0.52	23.87 ± 0.65	**32.12 ± 0.83**	27.13 ± 0.48	12.74 ± 0.19
( **2**)	2.71 ± 0.01	**8.07 ± 0.37**	21.31 ± 0.98	**26.66 ± 1.06**	**23.04 ± 0.23**	11.46 ± 0.07
Control ^d^	2.97 ± 0.01	11.96 ± 0.01	22.22 ± 0.02	29.67 ± 0.23	27.35 ± 0.17	11.78 ± 0.11

^a^ Working at the IC_50_ × 2 for each fraction; ^b^ The concentrations of the metabolites were calculated using DMF as the internal standard (for details see Experimental Section); ^c^ Statistically significant changes were observed (Student’s t test) for the signals in bold (*p* < 0.05); ^d^ Untreated parasite.

#### 2.2.2. Inhibition of Mitochondrial Reductases

**Figure 6:**
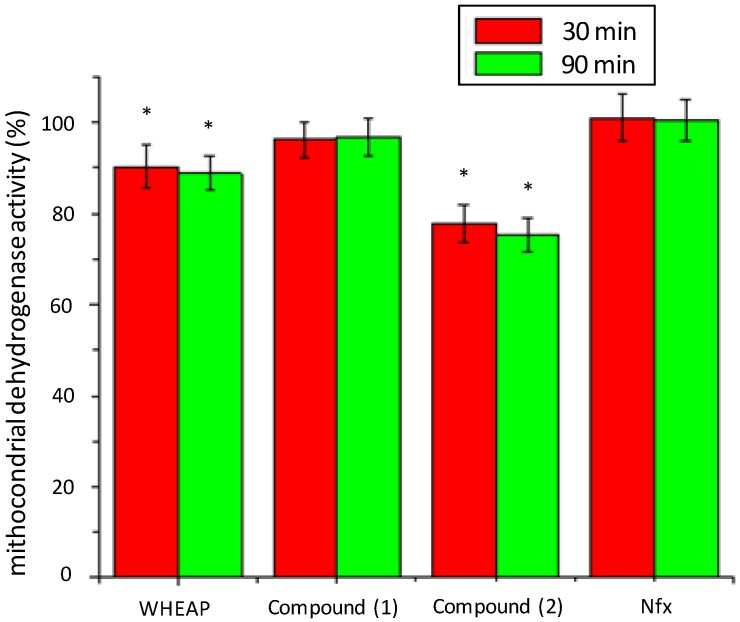
Mitochondrial dehydrogenases activities (%) compared to untreated control of *T. cruzi* epimastigote, Y strain, with time and compound. Bnz did not produce depletion of mitochondrial dehydrogenase activity at 20 μM in these conditions [21]. The experiment was done in duplicate. * Statistically different to untreated control (Student’s t test) (*p* < 0.05).

According to the results in the changes of excreted metabolites by action of the extract and isolated compounds, mitochondrial reductases can be proposed as their potential biological targets. For that reason, we analyzed the effect on these enzymes using a procedure that involves MTT exposure for short times [21,23]. This study is a colorimetric assay using MTT, which is enzymatically reduced to blue formazan. Short assay times, less than 4 h, it can demonstrate the activity of mitochondrial dehydrogenases while the microorganism is still viable. Under the assay conditions, after using MTT for short times to detect dehydrogenase activity, the specific enzymes that are evaluated are succinate and malate dehydrogenase, SDH and MDH, respectively. The metabolomic studies described showed that the metabolite excreted to a lesser extent compared to untreated parasite was Succ. Since mitochondrial SDH and fumarate reductases (FRDs) are enzymes endowed with a high degree of homology it is likely that most SDH inhibitors could also act by inhibiting FRDs, and according with our results (Figure 6) this could be the case with wheap and compound **2**. In trypanosomatids FRDs are located in at least two different compartments, namely the mitochondria and the glycosome, and dihydroorotate dehydrogenase (DHOD) could also act as a soluble FRD. The inhibition of the mitochondrial FRD promoted the depletion of fumarate with concomitant decrease in Succ production. On the other hand inhibition of glycosomal FRD or DHOD should also lead to a decrease in Succ excretion, consequently an action of the compounds at these levels could not be excluded [20].

#### 2.2.3. Inhibition of Membrane Sterol Biosynthesis

**Figure 7:**
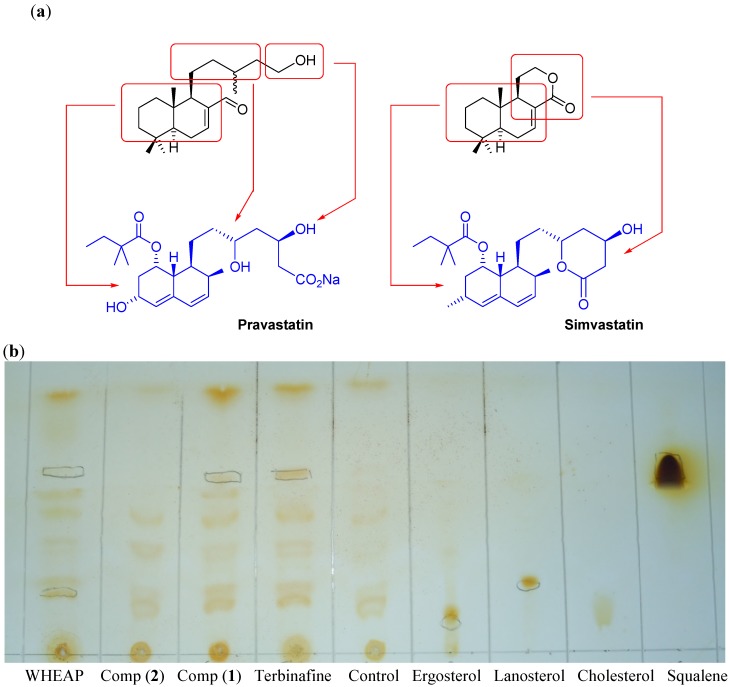
(**a**) Structure of some statins, *i.e.*, pravastatin and simvastatin, structurally related to compounds **1** and **2**. (**b**) Examples of TLCs of the study of changes in the membrane sterols. Control means untreated parasite. Terbinafine was used as control of *T. cruzi* squalene accumulator.

Trypanosomatids resemble fungi in terms of their endogenous biosynthesis and cellular sterol composition [24]. Unlike mammals, which synthesize cholesterol, epimastigote forms of *T. cruzi* produce mostly ergosterol [25]. *T. cruzi* contains significant amounts of cholesterol of exogenous origin [26], mostly in amastigotes [27], but it remains highly susceptible to sterol biosynthesis inhibitors, demonstrating a need for specific sterols not synthesized by the host [28]. The sterol biosynthesis pathway is therefore considered one of the most attractive targets for the specific treatment of Chagas disease [29,30], and several enzymes from this pathway have been studied as possible treatment targets [31]. Some of the compounds able to inhibit the sterol biosynthesis are the well-known statins which are used in the treatment of cholesterol-enhanced diseases [32]. From a structural point of view, some statins are alcohol or lactone containing decalins, *i.e.*, pravastatin and simvastatin (Figure 7a). This structural framework resembles the structure of the active labdanes **1** and **2**. For that reason, we analyzed the capacity of the extract and the active compounds to inhibit any of the enzymes involved in the biosynthesis of membrane sterols using the analysis of the accumulation or depletion of some intermediates or final product of this biochemical pathway. Qualitative analyses were performed by thin layer chromatography (TLC). In this experiment compound **1** and wheap were able to accumulate squalene and deplete ergosterol (Figure 7b) as did the positive control terbinafine, a well-known antifungal with anti-*T. cruzi* activity. In this way a possible target might be the enzyme squalene-2,3-epoxidase, which catalyzes the conversion of squalene into lanosterol [30]. Additionally, wheap and compound **1** accumulated lanosterol and compound **2**, compared to control, did not show any changes in the levels of studied sterols and intermediates.

## 3. Experimental Section

### 3.1. In Vitro Anti-trypomastigotes Assay

For assays with *T. cruzi* trypomastigotes, blood parasites were maintained by successive passages every seven days in female and male BALB/c mice. For this purpose, the infected mice were euthanized, the blood parasites content were counted and healthy mice were reinfected to maintain the parasites. In the assay 96-well plates were used, in which the mouse blood containing parasites at a concentration of one million per mL, in a ratio 90/10 respect to compounds, was placed. For the negative control phosphate buffered saline (PBS) was used and mouse blood containing parasites, and gentian violet (GV was used) for the positive control. The parasites were incubated at 4 °C for 24 h and then was performed the observation and counting of parasite contents of each well. The percentage of lyses was calculated using the following formula: % lyses = (number of parasites in untreated control—number of parasites with compound)/(number of parasites in untreated control) × 100 [17]. The experiment was done in duplicate.

### 3.2. In Vivo Studies

#### 3.2.1. Mice and Parasites

Female and male BALB/c mice were bred at the “Instituto de Investigaciones en Ciencias de la Salud” IICS (Asunción, Paraguay) and were 6–8 weeks of age when used. In this experiment, the clone CL Brener of *Trypanosoma cruzi* supplied by Dr. B. Zingales, Sao Paulo, Brazil was used [33]. Routine maintenance of the *Trypanosoma cruzi* strain was carried out in BALB/c mice inoculated by intraperitoneal via every 14 days. All mice were infected intraperitoneally with 5,000 blood trypomastigotes of CL Brener clone.

#### 3.2.2. Treatment

The treatments were started 7 days after inoculation of the parasites. The mice were divided into groups of eight animals. Benznidazole (Bnz) (Roche, Basel, Switzerland) was selected as reference drug for the experiment. All the products evaluated were diluted in PBS—Tween 80 (4:1). Bnz was administered to BALB/c mice via oral in regimens of 50 mg/kg b.w. daily for 2 weeks. The hydro-ethanolic extract of aerial parts of *Aristeguietia glutinosa* was administered orally at 50 mg/kg b.w. daily for 2 weeks. Compound **1** was administered orally at 30 and 10 mg/kg b.w. daily for 2 weeks [34]. 

#### 3.2.3. Treatment Outcome

Parasitemias in control and treated mice were determined once weekly for 60 days in tail-vein blood and the mortality rate was recorded.

### 3.3. ^1^H-NMR Study of the Excreted Metabolites

For the ^1^H-NMR spectroscopic studies [17,20,25], 2-day-treated *T. cruzi* (Y strain), with each studied compound at concentrations corresponding to the 2 × IC_50_ values (5 mL) was centrifuged at 1500 *×g* for 10 min at 4 °C. The pellet was discarded, and the parasite-free supernatant was stored at −20 °C until use. Before measuring, DMF (0.1 mL, 10 mM) as the internal standard and D_2_O (0.1 mL) were added to the supernatant (0.3 mL). ^1^H-NMR experiments were recorded at 20 °C on a Bruker Avance DPX-400 spectrometer (Bruker, Rheinstetten, Germany), operating at 400.132 MHz, with a 5 mm broadband inverse geometry probe. The acquisition parameters included: 90° pulse (zgpr, avance-version v 1.7.10.2, 1D sequence with f1 presaturation), 128 scans, and spectral width of 14.983 ppm. The acquisition time was 1.3664 s. Signal intensities were calculated by performing appropriate baseline corrections and then integrating the area under each of the resonances using MestRe-C NMR software [35]. Spectra were analyzed using the Topspin 1.3 software package. The spectra were registered with water suppression in 5 mm NMR (Aldrich, St. Louis, MO, USA) sample tubes. The chemical displacements used to identify the respective metabolites were previously confirmed by adding each analyzed metabolite to the studied supernatant as well as by the study of a control solution with 4 μg/mL of each metabolite in buffer (phosphate, pH = 7.4). Each run was done at least in triplicate and the Student t test was used to analyse the significance of the changes. The chemical shifts (δ, ppm) and multiplicity of the analysed catabolites were: Ala (alanine), 1.316, d; Lac (lactate), 1.466, d; Ace (acetate), 1.904, s; Pyr (pyruvate), 2.357, s; Succ (succinate), 2.392, s; Gly (glycine), 3.547, s [25].

### 3.4. Reductases Mitochondrial Inhibition Assay

Mitochondrial dehydrogenase activities were measured in 24-well plates. One million *T. cruzi* epimastigotes (Y strain) in 600 µL medium were seeded in each well, and 5 µL of studied compounds was added. The WHEAP and compounds **1**, **2** and Nfx were evaluated at their respectively IC_50_ values. The IC_50_ of Nfx was 9.7 μM. Two wells with untreated parasites were maintained as controls corresponding to the given time of treatment. The cultures were incubated at 28 °C. At the different time incubations, the epimastigotes were counted, and the colorimetric MTT dye-reduction assay was performed, the tetrazolium salt being converted into purple formazan by mitochondria. Twenty five microlitres of a solution containing 4 mg/mL of MTT in PBS were added to each well, and plates were incubated for an additional 2 h. The reaction was stopped by addition of 100 µL of solution SDS–isopropanol (10% SDS, 50% isopropanol, 40% water). The absorbance was measured at 570 nm. Under our conditions, compounds did not interfere with the reaction mixture. Percentage of mitochondrial dehydrogenase activities (%) was determined using untreated parasites-activities as 100%. The experiment was done in duplicate. Results are expressed as the mean ± standard error of the mean (SEM), and comparisons were made using Student’s t test. A probability of 0.05 or less was considered significant [17,25].

### 3.5. Inhibition of Membrane Sterols Biosynthesis

Compounds with the parasite were incubated, starting from a parasitic load of 10 million per mL, for 72 h at 28 °C at their respective IC_50_. The parasites, epimastigotes of Y strain of *T. cruzi*, were grown in 6 mL of BHT medium in culture bottles. A negative control is performed with parasites in the absence of compounds. The positive control is done with terbinafine (IC_50_ = 44.7 μM) which has proven inhibitory activity on membrane sterol biosynthesis. On completion of incubation, the extraction of membrane sterols was performed. For this purpose the content of the culture bottle was centrifuged at 3,000 rpm for 10 min, the supernatant was discarded and the pellet was suspended in sodium phosphate buffer solution (6 mL, 0.05 M, pH 7.4). Then were centrifuged again at 3,000 rpm for 10 min and the supernatant was discarded. The resulting pellet was resuspended in a chloroform/methanol (2:1) mixture (5 mL) and the suspension was kept at 4 °C for 12 h. Then saturated NaCl solution (5 mL) was added and the mixture extracted once with chloroform (3 mL) and once with hexane (3 mL) with care to avoid taking any aqueous phase. The extracted volume was applied on a silica gel TLC plate. Chromatography was performed eluting with hexane, two runs to identify squalene, and once with hexane/EtOAc (8:2) for ergosterol. The plate was revealed under ultraviolet light or by exposure to iodine vapors and later burned. The iodine detection was specific of squalene (wine color spot). Also controls and commercial samples of ergosterol, lanosterol, cholesterol and squalene were run on the TLC plate [36]. The experiment was done in triplicate.

## 4. Conclusions

The *in vivo* results observed with this plant extract at the dose used in this study and the lack of mutagenic effects encourages continuing the development of this product by completing the safety and pharmacology profile. The research and development of compounds of high efficiency and low cost is extremely necessary for the future of the Chagas-infected population in Latin America. We have shown a possible mechanism of action of the test compounds involving inhibition of the activity of the mitochondrial dehydrogenases of the parasite for compound **2**, and inhibition of the biosynthesis of membrane sterols of *T.* *cruzi* for compound **1**, while wheap shares both mechanism of action as it contains both active metabolites, which could explain the *in vivo* activity of this extract.

## References

[1] Maya J.D., Orellana M., Ferreira J., Kemmerling U., López-Muñoz R., Morello A. (2010). Chagas disease: Present status of pathogenic mechanisms and chemotherapy. Biol. Res..

[2] WHO, GHO (2011). World Health Organization: Global Health Observatory Data Repository.

[3] Barrett M.P., Croft S.L. (2012). Management of trypanosomiasis and leishmaniasis. Br. Med. Bull..

[4] Tekiel V., Alba-Soto C.D., González Cappa S.M., Postan M., Sánchez D.O. (2009). Identification of vaccines candidates against Trypanosoma cruzi by immunization with sequential fractions of an epimastigote-subtracted trypomastigote cDNA expression library. Vaccine.

[5] Cerecetto H., González M. (2010). Synthetic medicinal chemistry in chagas’ disease: Compounds at the final stage of “hit-to-lead” phase. Pharmaceuticals.

[6] King R.M., Robinson H. (1975). Studies in the Eupatorieae (Asteraceae), CXXXIX. A new genus, *Aristeguietia*. Phytologia.

[7] Duke J.A., Bogenschutz-Godwin M.J., Ottesen A.R. (2008). Duke’s Handbook of Medicinal Plants of Latin America.

[8] Sharma O.P., Dawra R.K., Kurada N.P., Sharma P.D. (1998). A review of the toxicosis and biological properties of the genus *Eupatorium*. Nat. Toxins.

[9] Woerdenbag H.J. (1986). *Eupatorium cannabinum* L. A review emphasizing the sesquiterpene lactones and their biological activity. Pharm. Weekbl. Sci. Ed..

[10] Acosta-Solis M. (1992). Vademecum de Plantas Medicinales del Ecuador.

[11] Valencia R., Pitman N., León-Yánez S., Jorgensen P.M. (2000). Libro Rojo de Las Plantas Endémicas del Ecuador.

[12] El-Seedi H.R., Ohara T., Sata N., Nishiyama S. (2002). Antimicrobial diterpenoids from *Eupatorium glutinosum* (Asteraceae). J. Ethnopharmacol..

[13] El-Seedi H.R., Sata N., Torssell K.B.G., Nishiyama S. (2002). New labdene diterpenes from Eupatorium glutinosum. J. Nat. Prod..

[14] Abad M.J., Bermejo P., Sanchez Palomino S., Chiriboga X., Carrasco L. (1999). Antiviral activity of some South American medicinal plants. Phytother. Res..

[15] Chiriboga X., Miño P. (2011). Antimicrobial activity of *Aristeguietia glutinosa*.

[16] Varela J., Lavaggi M.L., Cabrera M., Rodríguez A., Miño P., Chiriboga X., Cerecetto H., González M. (2012). Bioactive-guided identification of labdane diterpenoids from aerial parts of *Aristeguietia glutinosa* Lam. as anti-*Trypanosoma cruzi* agents. Nat. Prod. Commun..

[17] Benitez D., Cabrera M., Hernández P., Boiani L., Lavaggi M.L., di Maio R., Yaluff G., Serna E., Torres S., Ferreira M.E. (2011). 3-Trifluoromethylquinoxaline *N*,*N*'-dioxides as anti-trypanosomatid agents. Identification of optimal anti-*T. cruzi* agents and mechanism of action studies. J. Med. Chem..

[18] Sánchez-Moreno M., Fernandez-Becerra M.C., Castilla-Calvente J.J., Osuna A. (1995). Metabolic studies by ^1^H NMR of different forms of *Trypanosoma cruzi* as obtained by “*in vitro*” culture. FEMS Microbiol. Lett..

[19] Mesa-Valle C.M., Castilla-Calvente J., Sánchez-Moreno M., Moraleda-Lindez V., Barbe J., Osuna A. (1996). Activity and mode of action of acridine compounds against Leishmania donovani. Antimicrob. Agents. Chemother..

[20] Boiani L., Aguirre G., González M., Cerecetto H., Chidichimo A., Cazzulo J.J., Bertinaria M., Guglielmo S. (2008). Furoxan-, alkylnitrate-derivatives and related compounds as anti-trypanosomatid agents: Mechanism of action studies. Bioorg. Med. Chem..

[21] Boiani M., Boiani L., Merlino A., Hernández P., Chidichimo A., Cazzulo J.J., Cerecetto H., González M. (2009). Second generation of 2*H*-benzimidazole 1,3-dioxide derivatives as anti-trypanosomatid agents: Synthesis, biological evaluation, and mode of action studies. Eur. J. Med. Chem..

[22] Bringaud F., Riviere L., Coustou V. (2006). Energy metabolism of trypanosomatids: Adaptation toavailable carbon sources. Mol. Biochem. Parasitol..

[23] Sánchez-Moreno M., Gómez-Contreras F., Navarro P., Marín C., Ramírez-Macías I., Olmo F., Sanz A.M., Campayo L., Cano C., Yunta M.J. (2012). *In vitro* leishmanicidal activity of imidazole- or pyrazole-based benzo[*g*]phthalazine derivatives against *Leishmania infantum* and *Leishmania braziliensis* species. J. Antimicrob. Chemother..

[24] Fernández M., Varela J., Correia I., Birriel E., Castiglioni J., Moreno V., Costa Pessoa J., Cerecetto H., González M., Gambino D. (2013). A new series of heteroleptic oxidovanadium (IV) compounds with phenanthroline-derived co-ligands: Selective *Trypanosoma cruzi* growth inhibitors. Dalton Trans..

[25] Benítez D., Casanova G., Cabrera G., Galanti N., Cerecetto H., González M. (2014). Initial studies on mechanism of action and cell death of active *N*-oxide-containing heterocycles in *Trypanosoma cruzi* epimastigotes *in vitro*. Parasitology.

[26] Roberts C.W., McLeod R., Rice D.W., Ginger M., Chance M.L. (2003). Fatty acid and sterol metabolism: Potential antimicrobial targets in apicomplexan and trypanosomatid parasitic protozoa. Mol. Biochem. Parasitol..

[27] Urbina J.A., Vivas J., Visbal G., Contreras L.M. (1995). Modification of the sterol composition of *Trypanosoma* (Schizotrypanum) *cruzi* epimastigotes by delta 24(25)-sterol methyl transferase inhibitors and their combinations with ketoconazole. Mol. Biochem. Parasitol..

[28] Liendo A., Visbal G., Piras M.M., Piras R., Urbina J.A. (1999). Sterol composition and biosynthesis in *Trypanosoma cruzi* amastigotes. Mol. Biochem. Parasitol..

[29] De Souza W., Rodrigues J.C. (2009). Sterol biosynthesis pathway as target for antitrypanosomatid drugs. Interdiscip. Perspect. Infect. Dis..

[30] Urbina J.A. (2009). Ergosterol biosynthesis and drug development for Chagas disease. Mem. Inst. Oswaldo Cruz.

[31] Kessler R.L., Soares M.J., Probst C.M., Krieger M.A. (2013). *Trypanosoma cruzi* Response to Sterol Biosynthesis Inhibitors: Morphophysiological Alterations Leading to Cell Death. PLoS One.

[32] Cabral M.E., Figueroa L.I., Fariña J.I. (2013). Synergistic antifungal activity of statin-azole associations as witnessed by Saccharomyces cerevisiae- and Candida utilis-bioassays and ergosterol quantification. Rev. Iberoam. Micol..

[33] Cano M.I., Gruber A., Vazquez A.M., Cortés A., Levin M.J., González A., Degrave W., Rondinelli E., Zingales B., Ramirez J.L. (1995). Molecular karyotype of clone CL Brener chosen for the *Trypanosoma cruzi* genome project. Mol. Biochem. Parasitol..

[34] Ferreira M.E., Cebrián-Torrejón G., Segovia Corrales A., Vera de Bilbao N., Rolón M., Vega Gomez C., Leblanc K., Yaluff G., Schinini A., Torres S. (2011). *Zanthozylum chiloperone* leaves extract: First sustainable Chagas disease treatment. J. Ethnopharmacol..

[35] Mestrelab Research. http://mestrelab.com.

[36] Gerpe A., Alvarez G., Benítez D., Boiani L., Quiroga M., Hernández P., Sortino M., Zacchino S., González M., Cerecetto H. (2009). 5-Nitrofuranes and 5-nitrothiophenes with anti-*Trypanosoma cruzi* activity and ability to accumulate squalene. Bioorg. Med. Chem..

